# Artificial Intelligence Chatbot Dependence, Anxiety, and Depressive Symptoms in University Students: Initial Psychometric Evaluation of the Artificial Intelligence Chatbot Dependence Scale (AICDS) in Spanish

**DOI:** 10.31083/AP53298

**Published:** 2026-08-20

**Authors:** Iván Barrios, Julio Torales, Sandra Blanco, Carlos Manuel Giménez-Barboza, Brenda Mikaela Monges-Guerrero, Axel Verón-Otero, Gladys Mercedes Estigarribia Sanabria, Eva Natalia Giménez-Legal, João Mauricio Castaldelli-Maia, Antonio Ventriglio

**Affiliations:** ^1^Cátedra de Metodología de la Investigación II, Filial Santa Rosa del Aguaray, Facultad de Ciencias Médicas, Universidad Nacional de Asunción, 111421 Santa Rosa del Aguaray, Paraguay; ^2^Faculty of Health Sciences, Universidad Sudamericana, 7800 Salto del Guairá, Paraguay; ^3^Grupo de Investigación sobre Epidemiología de los Trastornos Mentales, Psicopatología y Neurociencias, Facultad de Ciencias Médicas, Universidad Nacional de Asunción, 111421 San Lorenzo, Paraguay; ^4^Vicerrectoría de Investigación y Postgrado, Universidad de Los Lagos, 5311157 Osorno, Chile; ^5^Department of Psychiatry, University of São Paulo, São Paulo, SP 05403-903, Brazil; ^6^Department of Clinical and Experimental Medicine, University of Foggia, 71122 Foggia, Italy

**Keywords:** artificial intelligence, chatbots, anxiety, depressive symptoms, university students, mental health

## Abstract

**Background:**

Artificial intelligence (AI) chatbots are rapidly becoming integrated into higher education, supporting human–computer interaction, knowledge explanation, rapid access to information, access to online learning resources, and personalized learning pathways. However, little is known about potential patterns of dependence on these technologies and their relationship with anxiety and depressive symptoms. This study aimed to conduct an initial psychometric evaluation of the Spanish version of the Artificial Intelligence Chatbot Dependence Scale (AICDS) and to explore its association with anxiety and depressive symptoms among university students.

**Methods:**

A cross-sectional study was conducted among 200 university students from Santa Rosa del Aguaray, Paraguay. Participants completed the AICDS together with screening measures of anxiety and depressive symptoms. Data were analyzed using R version 4.5.3. Confirmatory factor analysis using diagonally weighted least squares estimation with robust standard errors was performed to examine the scale's factor structure, and multiple linear regression models were used to evaluate associations between AICDS scores and anxiety and depressive symptoms.

**Results:**

The findings supported a unidimensional factor structure with acceptable-to-good model fit and high internal consistency (Cronbach's α = 0.833; McDonald's ω = 0.903). Fully standardized factor loadings ranged from 0.538 to 0.873. In bivariate analyses, students who screened positive for anxiety symptoms had higher AICDS scores than those who screened negative, although the effect size was small. However, this association was not maintained after adjustment for sociodemographic and academic covariates in multiple regression models. No significant association was observed between AICDS scores and depressive symptoms under two-tailed testing.

**Conclusions:**

These findings provide preliminary evidence supporting the internal consistency and factorial validity of the Spanish version of the AICDS in this sample. However, associations between AI chatbot dependence and anxiety or depressive symptoms were weak and not robust after adjustment. Further longitudinal and psychometric research is needed before broader conclusions can be drawn about the mental health implications of AI chatbot dependence among university students.

## Main Points

1. The Spanish version of the Artificial Intelligence Chatbot Dependence Scale (AICDS) showed a coherent unidimensional structure and high internal consistency in this sample.

2. AICDS scores were higher among students screening positive for anxiety symptoms in bivariate analyses, but this association was small and did not remain significant after adjustment for sociodemographic and academic covariates.

3. No statistically significant association was found between AICDS scores and depressive symptoms under two-tailed testing.

4. These findings support the AICDS as a promising instrument for further research, but additional validation studies with larger and more diverse samples are needed.

## 1. Introduction

The rapid expansion of artificial intelligence (AI) technologies has transformed multiple domains of everyday life, including higher education. Among these innovations, AI-based conversational agents, commonly referred to as chatbots, have become increasingly integrated into the academic routines of university students. These systems allow users to interact through natural language and obtain immediate responses to questions, explanations of complex topics, and assistance in completing academic tasks [1,2]. As a result, AI chatbots are progressively being incorporated into study practices, information-seeking behaviors, and learning processes in higher education environments [3,4,5,6].

The growing reliance on AI chatbots among university students represents an emerging phenomenon that may extend beyond purely instrumental academic use. Chatbots are computer programs designed to simulate conversation with human users through natural language interaction [7]. In the present study, chatbot dependence was understood as the tendency to rely on conversational AI systems for academic support, social interaction, or emotional reassurance in situations involving uncertainty or stress. Although the concept of “digital natives” has been debated, contemporary university students are frequently exposed to digital technologies and may rapidly incorporate emerging tools, including AI chatbots, into their academic and everyday practices [1,8].

Importantly, dependence on AI chatbots should be distinguished from frequent or academically appropriate use. In university settings, students may use chatbots as legitimate learning aids for clarifying concepts, organizing information, obtaining feedback, supporting writing-related tasks, or facilitating self-directed study [1,3,6]. Such use does not necessarily imply problematic behavior. Building on this literature, the present study operationalizes chatbot dependence as a pattern of perceived reliance, difficulty functioning without chatbot access, excessive trust in chatbot outputs, and repetitive consultation that may go beyond instrumental academic support. This distinction is important to avoid pathologizing ordinary AI-assisted learning while still allowing empirical examination of potentially maladaptive patterns of use.

University life is widely recognized as a period characterized by multiple developmental and psychosocial challenges. Students often face substantial academic demands, evolving social relationships, financial pressures, and the transition toward independent adulthood, all of which may significantly affect mental health [9,10]. A growing body of research has documented elevated levels of psychological distress among university students worldwide, including symptoms of anxiety, depression, and stress [11,12,13,14]. These concerns have become particularly salient in recent years due to the disruptions caused by the Coronavirus disease 2019 (COVID-19) pandemic, which intensified students’ reliance on digital learning environments and online communication technologies [15,16,17].

Recent studies have begun to explore the potential relationship between the use of AI technologies and mental health outcomes among students. Some research suggests that intensive engagement with digital platforms and online tools may be associated with psychological distress, sleep disturbances, and problematic technology use [18,19,20]. Although AI chatbots differ from traditional social media or internet platforms, their conversational and interactive nature may encourage frequent use and emotional reliance. For example, students may use chatbots not only to obtain academic assistance but also to manage uncertainty, seek reassurance, or cope with academic stress.

Previous research on technology-related dependence has shown that problematic patterns of internet, smartphone, and social media use are frequently associated with depressive symptoms among university students. These associations may be explained by several mechanisms, including reduced sleep quality, displacement of offline social interaction, repetitive reassurance seeking, impaired self-regulation, and the use of digital tools as avoidant coping strategies [18,19,20,21]. Although AI chatbots differ from earlier digital platforms because they provide interactive, generative, and personalized responses, this literature offers a useful framework for examining whether dependence-like patterns of chatbot use may be related to depressive symptoms.

From a psychological perspective, digital technologies can function as mechanisms of cognitive and emotional regulation. Immediate access to information and feedback may help individuals reduce uncertainty and alleviate anxiety associated with complex or demanding tasks [22]. In academic contexts, AI chatbots may therefore operate as tools that facilitate rapid problem-solving while simultaneously providing a form of psychological reassurance. However, excessive reliance on such technologies could potentially contribute to maladaptive patterns of technology use or dependence, particularly if students begin to substitute independent problem-solving or interpersonal interactions with automated systems.

Another important concept in this context is cognitive offloading, which refers to the tendency to rely on external technological systems to support cognitive processes such as memory, reasoning, or decision-making [23,24]. AI chatbots may facilitate this process by allowing users to quickly outsource cognitive effort, which could increase efficiency but also encourage habitual reliance on digital assistance. Over time, this reliance may contribute to patterns of dependence that are conceptually related to other forms of problematic technology use.

Despite the growing interest in the educational and psychological implications of AI chatbots, empirical research examining the potential relationship between chatbot dependence and mental health remains limited. Furthermore, the systematic measurement of this construct is still in an early stage of development. Reliable and valid psychometric instruments are essential for advancing research in this area and for identifying potential risk factors associated with problematic technology use.

One instrument recently developed to address this gap is the Artificial Intelligence Chatbot Dependence Scale (AICDS), which was designed to assess psychological and behavioral dependence on AI chatbots in everyday life [25]. The scale evaluates dimensions such as excessive trust in chatbot responses, compulsive use, and perceived difficulty functioning without access to AI conversational systems. Initial validation studies have reported promising psychometric properties, suggesting that the AICDS may represent a useful tool for examining emerging patterns of technology dependence.

However, to date there are no validated Spanish-language versions of this instrument available for use in university populations. The absence of culturally adapted tools limits the ability to investigate chatbot dependence and its potential psychological consequences in specific Spanish-speaking university populations. Cross-cultural adaptation and psychometric validation are therefore necessary steps to ensure that the scale can be reliably used in different linguistic and cultural settings [26,27].

The marginal contribution of the present study is threefold. First, it provides an initial Spanish adaptation and psychometric evaluation of the AICDS in a South American university sample, addressing the lack of instruments for assessing AI chatbot dependence in Spanish-speaking contexts. Second, it examines whether AICDS scores are associated with anxiety and depressive symptoms using both bivariate and adjusted analytical approaches. Third, it contributes conceptually by distinguishing ordinary AI-assisted learning from dependence-like patterns of perceived reliance, excessive trust, and difficulty functioning without chatbot access. In this way, the study aims to support a more nuanced understanding of AI use in higher education without pathologizing legitimate academic engagement with these tools.

Given the increasing integration of AI technologies into students’ academic lives and the potential implications for psychological well-being, further research is needed to better understand how dependence on AI chatbots may relate to mental health outcomes. Therefore, the primary aim of the present study was to conduct an initial psychometric evaluation of the Spanish version of the AICDS, while a secondary aim was to examine its association with anxiety and depressive symptoms in a sample of university students from Santa Rosa del Aguaray, Paraguay. The following sections describe the study design, sampling strategy, measures, statistical analyses, psychometric results, associations with anxiety and depressive symptoms, and the implications and limitations of the findings.

## 2. Materials and Methods

### 2.1 Study Design

A quantitative observational study with a correlational scope and a cross-sectional design was conducted to examine the relationship between anxiety and depressive symptoms and dependence on AI chatbots among university students. This design allowed the characterization of associations between mental health variables and chatbot dependence at a specific point in time [28]. Because of its cross-sectional nature, the design did not permit causal inference or determination of the temporal direction between chatbot dependence and anxiety or depressive symptoms.

### 2.2 Population and Sampling

The target population comprised university students enrolled in undergraduate programs in Santa Rosa del Aguaray, Paraguay. This city represents an emerging regional educational center in northern Paraguay, attracting students from surrounding districts and rural areas. Consequently, although the study was conducted in a single city, the sample includes students from diverse socioeconomic and geographic backgrounds. The accessible population included students who had used an artificial intelligence chatbot within the previous four weeks.

Participants were recruited using a non-probabilistic consecutive sampling strategy based on an open invitation extended to students. This strategy enabled the inclusion of an accessible and heterogeneous sample in terms of academic level and individual characteristics. Eligibility criteria included being 18 years or older, currently enrolled in an undergraduate university program in Santa Rosa del Aguaray, having used an artificial intelligence chatbot during the previous four weeks, and voluntarily agreeing to participate in the study. Students on academic leave or temporary withdrawal were excluded prior to enrollment, and therefore no data were collected from these subgroups. Questionnaires with more than 20% missing responses were defined as ineligible; however, in practice all questionnaires were fully completed, and all collected responses were retained for analysis.

Data collection was carried out through an online questionnaire hosted on a freely accessible digital platform and disseminated through institutional channels, social media, and messaging applications such as WhatsApp and Telegram. The survey remained open from October to December 2025. Before accessing the survey, participants were informed about the objectives of the study, the voluntary nature of participation, and the anonymity and confidentiality of data handling. Online survey methods have been shown to be a methodologically valid and efficient strategy, producing results comparable to those obtained through traditional face-to-face surveys [29]. No incentives were offered for participation, and reminder messages were disseminated biweekly through the same institutional channels and social media platforms to encourage survey completion.

### 2.3 Variables

Several sociodemographic, academic, and psychological variables were collected to characterize the study population and explore their association with AI chatbot dependence. These variables were selected because sociodemographic and academic characteristics may influence both technology use and mental health, while anxiety and depressive symptoms represent common indicators of student psychological vulnerability and have been widely examined in relation to problematic digital technology use.

Sociodemographic variables included sex (male or female), age (in completed years), type of university (public or private), and academic field of study categorized into major disciplinary areas: natural sciences, engineering and technology, medical and health sciences, agricultural and veterinary sciences, social sciences, and humanities and arts.

Participants were also asked to report the primary purpose of their use of artificial intelligence chatbots through an open-ended question. Responses were coded into thematic categories after data collection. Two members of the research team independently reviewed the responses and proposed preliminary categories based on semantic similarity. These categories were then discussed and consolidated by consensus into five mutually exclusive groups: resolving academic doubts, writing assignments, curiosity or entertainment, emotional support, and other. Disagreements were resolved through discussion with a third researcher. Because the coding was descriptive and used only to characterize the sample rather than to test a primary hypothesis, no formal inter-rater reliability coefficient was calculated.

Mental health variables included symptoms of anxiety and depression. Anxiety was assessed using the Generalized Anxiety Disorder-7 scale (GAD-7), while depressive symptoms were measured using the Patient Health Questionnaire-2 (PHQ-2). The primary outcome variable of the study was dependence on artificial intelligence chatbots, measured using the AICDS.

### 2.4 Measures

#### 2.4.1 Artificial Intelligence Chatbot Dependence Scale (AICDS)

Dependence on AI chatbots was assessed using the AICDS, a self-report instrument designed to evaluate psychological and behavioral dependence on conversational artificial intelligence systems in everyday contexts. The scale explores patterns such as excessive trust in chatbot responses, compulsive consultation of AI systems, and perceived difficulty functioning without access to these tools.

The instrument consists of eight items organized in a unidimensional structure. Each item is rated using a seven-point Likert scale reflecting increasing levels of agreement or frequency. Higher scores indicate greater levels of dependence on AI chatbots.

Previous validation studies have reported adequate psychometric properties for the instrument, including a stable factor structure and approximately 58% of explained variance [25]. The scale does not include clinical cut-off points; therefore, scores are interpreted dimensionally using the total or average score as a continuous indicator of chatbot dependence.

##### Translation and Cultural Adaptation of the AICDS

The scale was translated and culturally adapted into Spanish following internationally recommended procedures for the cross-cultural adaptation of self-report instruments. The process included forward translation, back-translation, and review by bilingual researchers to ensure semantic and conceptual equivalence with the original instrument [26].

First, the original English version was independently translated into Spanish by two bilingual researchers familiar with psychological terminology. The two translated versions were then compared and synthesized into a preliminary Spanish version. Subsequently, this version was back translated into English by an independent bilingual translator who was blinded to the original instrument. The back-translated version was compared with the original scale to detect potential discrepancies and ensure conceptual equivalence. Minor wording adjustments were made to improve clarity and cultural appropriateness for Spanish-speaking university students. The final Spanish version of the scale was incorporated into the survey used in the present study.

The Spanish wording of the eight AICDS items used in this study is presented in the Results section together with the factor loadings.

#### 2.4.2 Patient Health Questionnaire-2 (PHQ-2)

Depressive symptoms were assessed using the PHQ-2, originally developed by Kroenke, Spitzer, and Williams (2003) [30]. The PHQ-2 comprises the first two items of the nine-item Patient Health Questionnaire (PHQ-9) and evaluates the presence of depressed mood and anhedonia during the previous two weeks.

Items are rated on a four-point Likert scale ranging from 0 (“not at all”) to 3 (“nearly every day”), resulting in a total score between 0 and 6. A score of 3 or higher has been proposed as a screening threshold for major depressive disorder, showing a sensitivity of approximately 83% and specificity of 92% [30]. The Spanish version of the PHQ-2 was used in the present study and has demonstrated adequate reliability in previous research [31].

#### 2.4.3 Generalized Anxiety Disorder-7 (GAD-7)

Anxiety symptoms were measured using the GAD-7, a widely used self-administered instrument designed to screen for generalized anxiety disorder and assess the severity of anxiety symptoms [32].

The scale consists of seven items rated on a four-point Likert scale ranging from 0 (“not at all”) to 3 (“nearly every day”), yielding a total score between 0 and 21. Cut-off scores of 5, 10, and 15 correspond to mild, moderate, and severe anxiety symptoms, respectively. A score of 10 or higher has been suggested as an appropriate threshold for identifying probable cases of generalized anxiety disorder. The validated Spanish version of the GAD-7 was used in this study and has demonstrated good psychometric properties in previous research [33].

### 2.5 Sample Size

The sample size was determined considering methodological recommendations for psychometric studies involving self-report instruments. Traditional guidelines suggest including between five and ten participants per item when conducting factor analyses, as well as minimum sample sizes ranging between 100 and 200 participants to obtain stable solutions [34]. Because the AICDS contains eight items, the minimum recommended sample size would range between 40 and 80 participants according to this rule of thumb. However, more recent methodological literature emphasizes the importance of overall sample size and model complexity in confirmatory factor analysis (CFA) rather than relying exclusively on item-based ratios. Studies on instrument validation commonly recommend samples of approximately 200 to 300 participants to obtain stable parameter estimates and reliable model fit indices [35,36,37]. In line with these recommendations, a total of 200 university students were included in the final sample, which is considered adequate for confirmatory factor analysis of relatively simple models with a limited number of items.

### 2.6 Statistical Analysis

Statistical analyses were conducted using R (version 4.5.3; R Foundation for Statistical Computing, Vienna, Austria; https://www.R-project.org/). Descriptive statistics were calculated to summarize sample characteristics. Quantitative variables were described using measures of central tendency and dispersion; categorical variables were presented as frequencies and percentages. Group comparisons were performed using Student’s *t*-tests or one-way analysis of variance (ANOVA), with a significance level of 5% (two-tailed for all tests). Effect sizes were estimated using Cohen’s d for *t*-tests and eta-squared (η^2^) for ANOVA, with 95% confidence intervals.

Psychometric analyses were conducted using the *lavaan* (version 0.6-21; https://CRAN.R-project.org/package=lavaan) and psych packages (version 2.6.3; https://CRAN.R-project.org/package=psych). The adequacy of the correlation matrix was assessed using the Kaiser–Meyer–Olkin (KMO) index and Bartlett’s test of sphericity. Polychoric correlations were computed for the ordinal item matrix, and item-level descriptive statistics and response frequency distributions were examined.

Confirmatory factor analysis was performed to evaluate the unidimensional structure of the AICDS. Because items were measured on an ordered Likert-type scale, the model was estimated using the diagonally weighted least squares (DWLS) method with robust standard errors (robust.sem parameterization), which is recommended for structural equation models with ordinal categorical indicators [38,39]. Model fit was evaluated using the Comparative Fit Index (CFI), Tucker–Lewis Index (TLI), Root Mean Square Error of Approximation (RMSEA) with 90% confidence interval, and Standardized Root Mean Square Residual (SRMR). Good fit was defined as CFI and TLI >0.95, RMSEA <0.05, and SRMR <0.08; acceptable fit as CFI and TLI between 0.90 and 0.95, RMSEA between 0.05 and 0.08, and SRMR between 0.05 and 0.10 [40]. Standardized residuals and modification indices were inspected to evaluate local model fit. Internal consistency was assessed using both Cronbach’s alpha (α) and McDonald’s omega (ω), the latter estimated via the psych package using polychoric correlations. Composite reliability (CR) and average variance extracted (AVE) were computed using semTools (version 0.5-8; https://CRAN.R-project.org/package=semTools) to provide additional evidence of construct reliability and item convergence within the latent factor.

To examine associations between chatbot dependence and mental health indicators, three complementary analytical approaches were employed. First, bivariate comparisons used independent-samples *t*-tests with Cohen’s d and 95% confidence intervals. Second, multiple linear regression models were fitted with GAD-7 and PHQ-2 continuous scores as predictors of AICDS total score, adjusting for age, sex, type of university, and field of study as potential confounders; standardized beta coefficients and 95% CIs are reported. Third, generalized additive models (GAMs) with thin-plate splines (k = 5) were used to test for nonlinear associations between anxiety and depressive symptom scores and chatbot dependence. Exploratory moderation analyses examined whether field of study, sex, or primary purpose of chatbot use moderated the association between anxiety symptoms and dependence. An exploratory mediation analysis with nonparametric bootstrap resampling (1000 iterations, bias-corrected and accelerated [BCa] 95% CI) tested whether anxiety (GAD-7) mediated the relationship between chatbot dependence and depressive symptoms (PHQ-2). Pearson and Spearman correlations between the AICDS total score and continuous GAD-7 and PHQ-2 scores were computed to explore associations with anxiety and depressive symptom severity. Variance inflation factors (VIF) were inspected to rule out multicollinearity in regression models.

### 2.7 Ethical Considerations

This study was conducted within the framework of the Scientific Research Initiation Program of the Research Group on Epidemiology of Mental Disorders, Psychopathology, and Neurosciences, and was approved by the Department of Research Methodology II of the School of Medical Sciences, National University of Asunción, Paraguay, in accordance with Resolution No. 0625-00-2025 of the Board of Directors of the School of Medical Sciences, Article 3, which regulates the ethical approval procedures for non-experimental research. Before accessing the questionnaire, participants were presented with an informed consent form describing the study objectives, voluntary nature of participation, estimated time required, anonymity, confidentiality, and absence of direct incentives. Consent was recorded electronically by selecting an acceptance option before proceeding to the survey. Participants could withdraw at any time before submitting their responses by closing the survey link, and no identifying information was collected. Data were stored in a password-protected digital file accessible only to the research team. Because the study included screening measures for anxiety and depressive symptoms, participants were informed that these instruments did not constitute a clinical diagnosis. Those who wished to receive individualized feedback or guidance were invited to contact the research team by email. Participants with elevated scores who requested feedback were advised to seek evaluation from qualified mental health professionals or appropriate university health services. Data were managed in accordance with the principles of confidentiality, equity, and justice, and in line with the ethical standards outlined in the Declaration of Helsinki.

## 3. Results

### 3.1 Sample Characteristics

A total of 200 university students were included in the study. Participants ranged in age from 18 to 55 years, with a mean age of 23.7 ± 6.1 years. Women represented 60.5% of the sample. Most participants were enrolled in public universities (66%), and half of the sample (50%) belonged to programs within the medical and health sciences. The most frequently reported use of artificial intelligence chatbots was resolving academic doubts (59.5%). Sociodemographic characteristics of the participants are presented in Table 1.

**Table 1.** **Sociodemographic and general characteristics of the participating students (N = 200)**.

Characteristic		n	%
Sex			
	Male	79	39.5
	Female	121	60.5
Type of university			
	Public	132	66.0
	Private	68	34.0
Field of study			
	Medical and health sciences	100	50.0
	Social sciences	43	21.5
	Natural sciences	19	9.5
	Engineering and technology	15	7.5
	Agricultural and veterinary sciences	15	7.5
	Humanities and arts	8	4.0
Primary purpose of AI chatbot use			
	Resolving academic doubts	119	59.5
	Writing assignments	32	16.0
	Curiosity or entertainment	20	10.0
	Emotional support	10	5.0
	Other	19	9.5

AI, artificial intelligence.

### 3.2 Psychometric Properties of the AICDS

The adequacy of the correlation matrix for factor analysis was supported by a high Kaiser–Meyer–Olkin index (KMO = 0.889) and a statistically significant Bartlett’s test of sphericity (χ^2^ = 728.90; df = 28; *p* < 0.001), indicating that the data were suitable for factor analysis.

A CFA was performed to test the unidimensional structure of the AICDS using DWLS estimation with robust standard errors. The one-factor model showed acceptable to good fit to the data: χ^2^(20) = 45.819; CFI = 0.994; TLI = 0.992; RMSEA = 0.081 (90% CI: 0.050–0.111); SRMR = 0.054. CFI and TLI values exceeded the 0.95 threshold for good fit. The RMSEA value of 0.081 falls within the acceptable range (0.05–0.10), and the SRMR of 0.054 indicates good residual fit. Standardized residuals were inspected; the largest off-diagonal values were observed for the AICDS6–AICDS7 pair (standardized residual = 4.49) and the AICDS1–AICDS7 pair (−4.87), and the top modification index suggested a correlated residual between AICDS6 and AICDS7 [modification index (MI) = 15.58, expected parameter change (EPC) = 0.21]. Given the absence of theoretical justification for these local dependencies, no modifications were introduced to the model; this pattern is noted as a psychometric limitation to be addressed in future studies with larger samples.

**Fig. 1.**
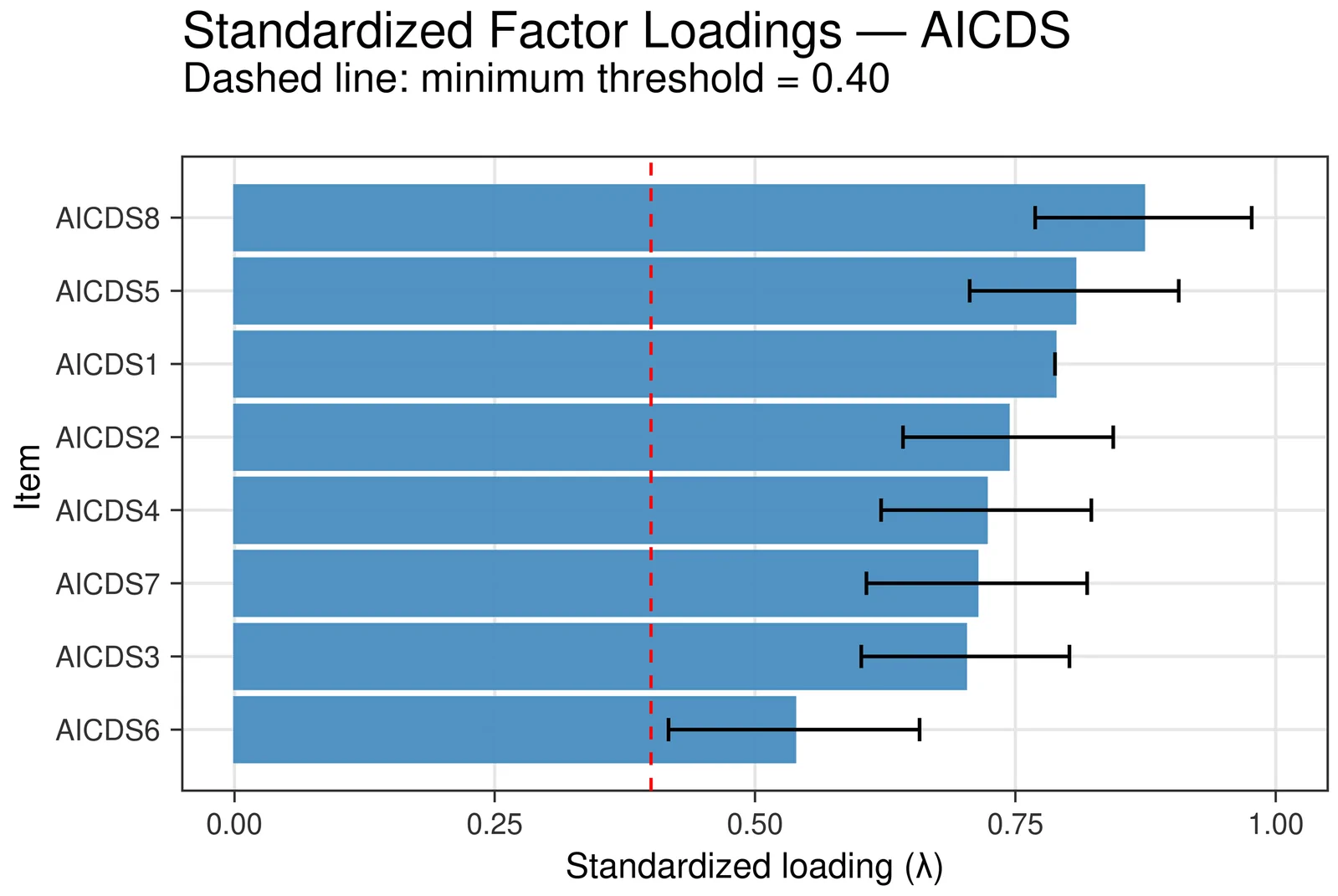
**Standardized factor loadings of the Spanish version of the AICDS estimated via DWLS**. Error bars represent 95% confidence intervals. The dashed red line indicates the minimum acceptable loading threshold of 0.40.

All factor loadings were statistically significant (*p* < 0.001). Fully standardized loadings ranged from 0.538 (AICDS6) to 0.873 (AICDS8), indicating moderate to strong relationships between each item and the latent construct (Table 2, Fig. 1).

**Table 2.** **Spanish item wording and standardized and unstandardized factor loadings of the Spanish version of the Artificial Intelligence Chatbot Dependence Scale (AICDS) (N = 200)**.

Item—Spanish wording	Unstandardized λ	SE	z	Standardized λ	Residual variance (1 − R^2^)
AICDS1—*Si no pudiera utilizar chatbots con IA*,* me sentiría ansioso/a o incómodo/a*.	1.000	—	—	0.788	0.379
AICDS2—*Necesito abrir los chatbots con IA antes de comenzar a trabajar o realizar mis tareas*.	0.943	0.052	18.253	0.743	0.448
AICDS3—*Si no pudiera utilizar chatbots con IA*,* me resultaría difícil obtener la información que necesito*.	0.891	0.051	17.381	0.702	0.508
AICDS4—*Incluso cuando me enfrento a tareas o trabajos que podría completar fácilmente por mi cuenta*,* tiendo a buscar ayuda en los chatbots con IA*.	0.916	0.052	17.663	0.722	0.479
AICDS5—*En comparación con otras personas o actividades*,* prefiero dedicar tiempo a utilizar chatbots con IA*.	1.024	0.051	19.972	0.807	0.349
AICDS6—*Aunque no esté utilizando activamente los chatbots con IA*,* suelo mantenerlos abiertos o funcionando en segundo plano*.	0.682	0.061	11.101	0.538	0.711
AICDS7—*Cada vez dedico más tiempo al uso de chatbots con IA*.	0.905	0.054	16.647	0.713	0.492
AICDS8—*Para mí*,* la vida sin chatbots con IA sería incómoda*.	1.108	0.053	20.931	0.873	0.238

DWLS estimation with robust standard errors. All loadings were significant at *p* < 0.001. λ, factor loading; SE, standard error. Standardized λ refers to the fully standardized loading. Residual variance was calculated as 1 − standardized λ^2^. Items were administered in Spanish and rated on a 7-point Likert scale ranging from 1 = strongly disagree to 7 = strongly agree. DWLS, diagonally weighted least squares. The Spanish wording of the AICDS items is presented in italics.

Internal consistency of the AICDS was high, with a Cronbach’s alpha coefficient of 0.833. The mean AICDS score was 21.84 ± 10.6, with observed values ranging from 8 to 55. McDonald’s omega (ω), estimated using polychoric correlations, was 0.903, consistent with Cronbach’s alpha and supporting high internal consistency under the unidimensional model. CR was 0.881 and AVE was 0.549, exceeding the 0.50 threshold commonly used to support item convergence within the latent construct.

Pearson and Spearman correlations were also examined to explore the relationship between AICDS total scores and continuous mental health scores. Pearson correlations were positive but small and non-significant: r = 0.073 (95% CI: −0.066 to 0.210, *p* = 0.304) with GAD-7, and r = 0.099 (95% CI: −0.040 to 0.235, *p* = 0.162) with PHQ-2. Spearman rank correlations were consistent: ρ = 0.059 (*p* = 0.404) and ρ = 0.085 (*p* = 0.234), respectively. These findings do not provide strong evidence of convergence with anxiety or depressive symptom severity. Instead, they suggest that AICDS scores may capture a behavioral pattern that is not fully explained by general symptoms of anxiety or depression in this sample. Further studies should examine convergent and discriminant validity using additional theoretically related and unrelated constructs.

Item-level descriptive statistics are presented in Table 3. All items showed right-skewed distributions (skewness range: 0.45–1.20), confirming that the use of DWLS estimation for ordinal data was appropriate. Mean item scores ranged from 2.37 (AICDS5) to 3.18 (AICDS6) on a 1–7 scale, indicating that most participants reported low to moderate levels of chatbot dependence on individual items. Response frequency distributions showed that the most frequent response category was 1 (“strongly disagree”) across all items, with between 31% and 42% of participants selecting this option.

**Table 3.** **Descriptive statistics of the Spanish version of the AICDS (N = 200)**.

Item	M	SD	Mdn	Min	Max	Skewness	Kurtosis
AICDS1	2.71	1.73	2	1	7	0.87	−0.23
AICDS2	2.52	1.68	2	1	7	1.03	−0.05
AICDS3	2.94	1.79	2	1	7	0.61	−0.76
AICDS4	2.94	1.95	2	1	7	0.69	−0.86
AICDS5	2.37	1.64	2	1	7	1.20	0.50
AICDS6	3.18	2.00	3	1	7	0.45	−1.18
AICDS7	2.68	1.75	2	1	7	0.80	−0.53
AICDS8	2.50	1.75	2	1	7	1.05	−0.03

N = 200. M, mean; SD, standard deviation; Mdn, median. All items were scored on a 7-point Likert scale ranging from 1 = strongly disagree to 7 = strongly agree.

**Fig. 2.**
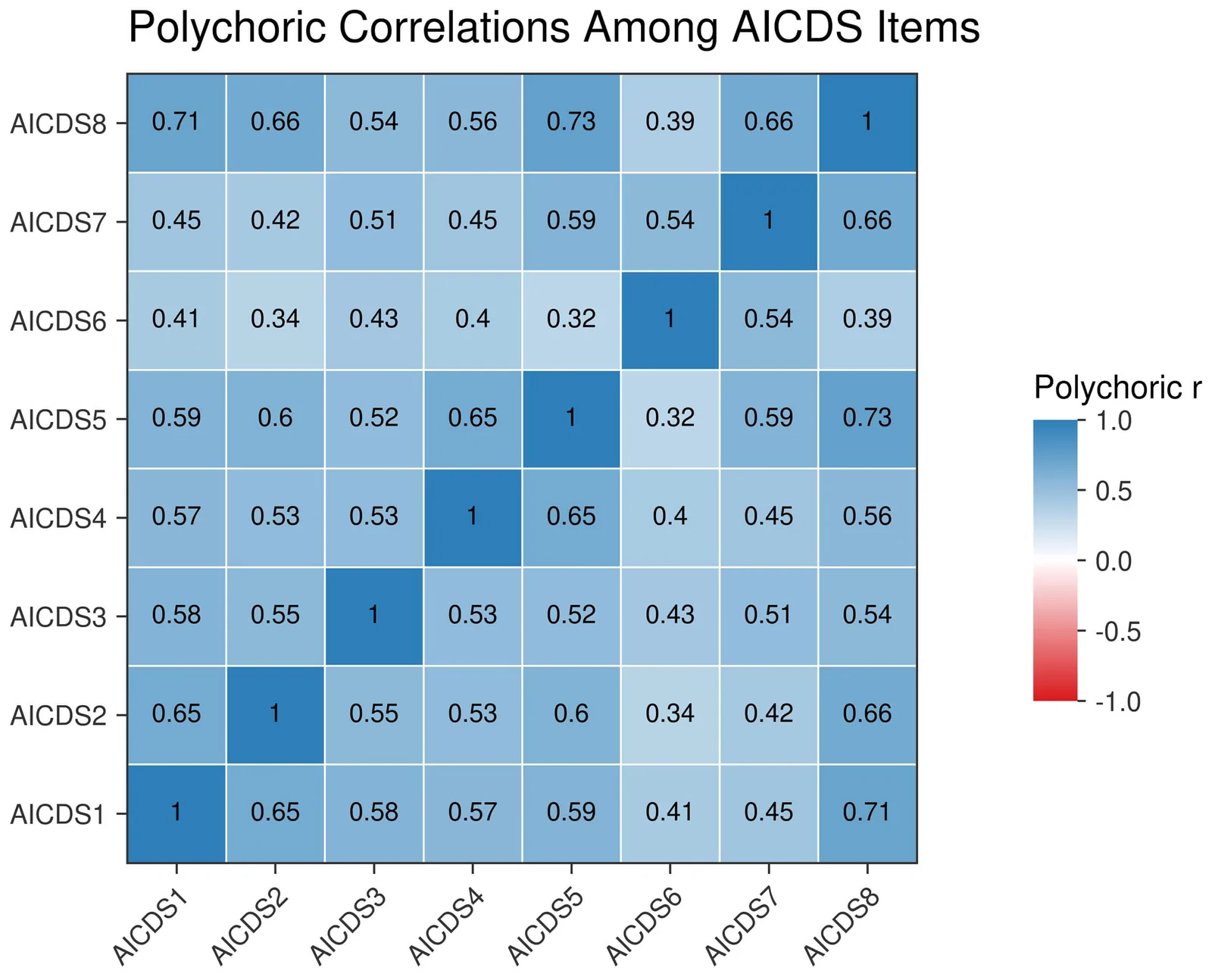
**Heatmap of polychoric correlations among the eight items of the Spanish version of the AICDS (N = 200)**. Color scale ranges from red (negative) to blue (positive). All inter-item correlations were positive, ranging from 0.32 to 0.73.

Polychoric correlations among AICDS items ranged from 0.323 (AICDS5–AICDS6) to 0.731 (AICDS5–AICDS8), with all inter-item correlations positive and moderate to strong in magnitude, supporting the internal coherence of the unidimensional structure (Fig. 2).

### 3.3 Mental Health Indicators

The mean GAD-7 score ranged from 0 to 21 points, with a mean of 8.3 ± 5.9. Based on the established cut-off criteria, 37.5% of participants screened positive for anxiety symptoms. The mean PHQ-2 score ranged from 0 to 6 points, with a mean of 2.26 ± 1.8, indicating that 42% of participants screened positive for depressive symptoms. Internal consistency for the GAD-7 and PHQ-2 scales was high, with Cronbach’s alpha coefficients of 0.905 and 0.746, respectively.

### 3.4 Associations Between Chatbot Dependence and Mental Health

Bivariate analyses showed no statistically significant differences in chatbot dependence scores according to sex or type of university (both *p* > 0.05). Similarly, no significant differences were observed across academic disciplines (ANOVA; all *p* > 0.05).

**Fig. 3.**
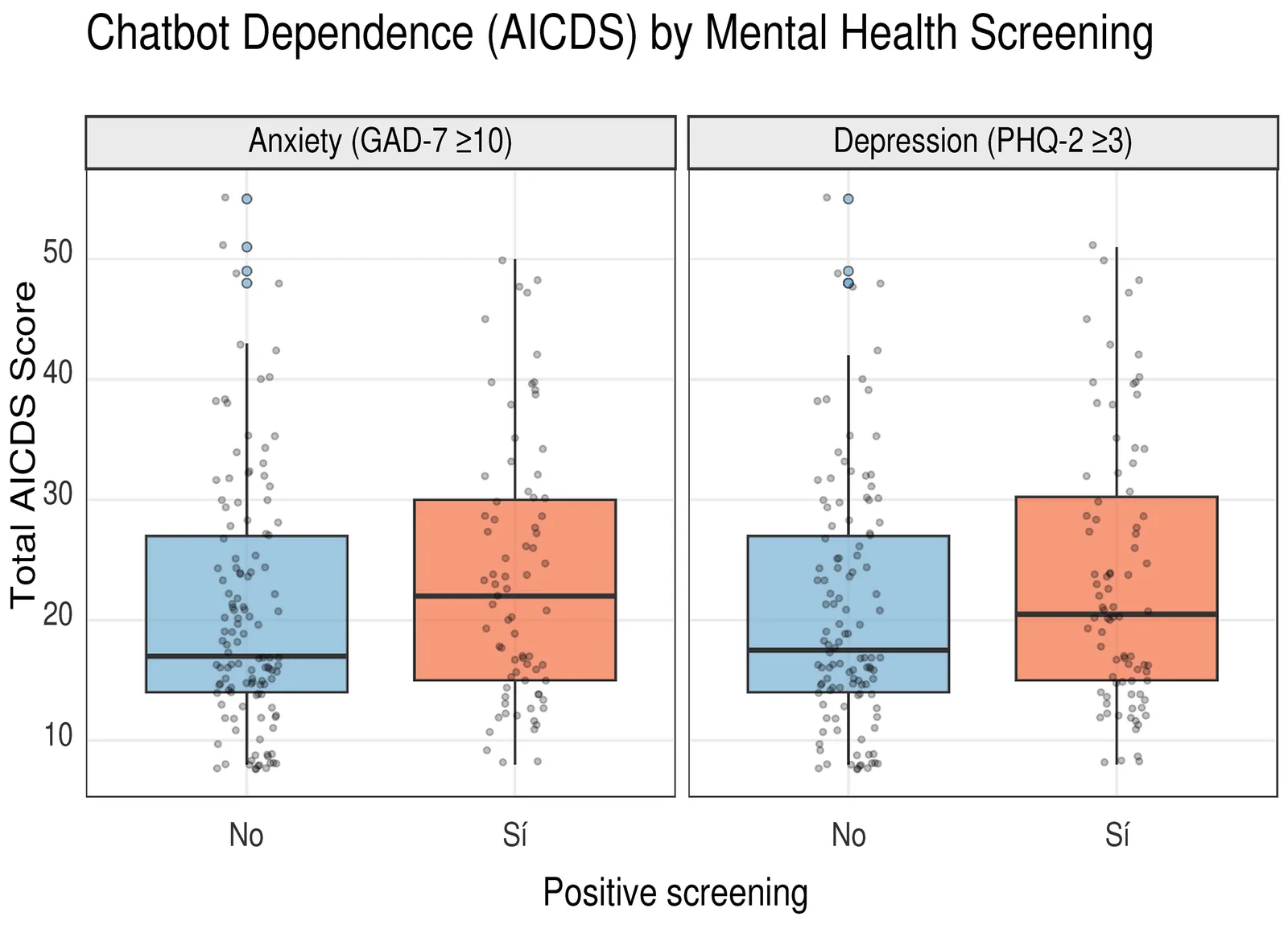
**Distribution of AICDS total scores by mental health screening status**. Boxplots show median, interquartile range, and individual data points (jittered). Left panel: anxiety screening (GAD-7 ≥10); right panel: depressive symptom screening (PHQ-2 ≥3). Screening-positive participants are shown in orange; screening-negative in blue. GAD-7, Generalized Anxiety Disorder-7 scale; PHQ-2, Patient Health Questionnaire-2.

In bivariate analyses, a statistically significant but small difference in chatbot dependence was observed according to anxiety screening status. Participants who screened positive for anxiety showed higher AICDS scores (M = 23.77, SD = 10.79) than those who screened negative (M = 20.68, SD = 10.49), with a mean difference of 3.09 points (95% CI: 0.06–6.12; *t*(198) = 2.01; *p* = 0.046). The effect size was small (Cohen’s d = −0.29, 95% CI: −0.58 to −0.01), with the negative sign reflecting the coding/order of group comparison rather than the direction described in the text. The comparison between participants screening positive and negative for depressive symptoms did not reach statistical significance under two-tailed testing (*t*(198) = −1.80, *p* = 0.073; mean difference = −2.72 points, 95% CI: −5.70 to 0.26; Cohen’s d = −0.26, 95% CI: −0.54 to 0.02) (Fig. 3).

To examine whether anxiety and depressive symptoms predicted chatbot dependence after controlling for potential confounders, multiple linear regression models were fitted with AICDS total score as the dependent variable. Model 1 included GAD-7 continuous score along with age, sex, type of university, and field of study as covariates. Model 2 replaced GAD-7 with PHQ-2 continuous score. Model 3 included both mental health scores simultaneously with all covariates (Table 4).

**Table 4.** **Multiple linear regression model predicting scores on the Spanish version of the AICDS (N = 200)**.

Predictor	B	SE	β	95% CI for B	*t*	*p*
GAD-7, continuous	0.05	0.17	0.03	[−0.28, 0.38]	0.30	0.763
PHQ-2, continuous	0.51	0.53	0.09	[−0.54, 1.55]	0.95	0.341
Age	−0.10	0.13	−0.06	[−0.36, 0.17]	−0.73	0.464
Sex, female	−1.78	1.62	−0.17	[−4.97, 1.41]	−1.10	0.272
University, private	1.20	1.71	0.11	[−2.18, 4.57]	0.70	0.485
Field: health sciences	−1.15	3.01	−0.11	[−7.10, 4.79]	−0.38	0.702
Field: natural sciences	−4.68	3.77	−0.44	[−12.12, 2.75]	−1.24	0.216
Field: social sciences	−1.18	3.28	−0.11	[−7.65, 5.28]	−0.36	0.719
Field: humanities	−7.62	4.97	−0.72	[−17.42, 2.18]	−1.53	0.127
Field: engineering	−5.15	3.96	−0.49	[−12.97, 2.67]	−1.30	0.196

This table presents Model 3, fully adjusted. Reference categories were male, public university, and agricultural/veterinary sciences. B, unstandardized coefficient; SE, standard error; β, standardized coefficient; CI, confidence interval. Model fit: R^2^ = 0.058, *F*(10, 189) = 1.17, *p* = 0.311.

In Model 1, the overall model was not statistically significant (*F*(9, 190) = 1.20, *p* = 0.295, R^2^ = 0.054). The GAD-7 score was not a significant predictor of chatbot dependence after adjustment (β = 0.08, 95% CI: −0.07 to 0.23, *p* = 0.276). Similarly, in Model 2, the PHQ-2 score did not significantly predict dependence when covariates were included (β = 0.10, 95% CI: −0.04 to 0.25, *p* = 0.157; *F*(9, 190) = 1.30, *p* = 0.240, R^2^ = 0.058). In the fully adjusted Model 3, neither GAD-7 (β = 0.03, 95% CI: −0.16 to 0.21, *p* = 0.763) nor PHQ-2 (β = 0.09, 95% CI: −0.09 to 0.27, *p* = 0.341) remained significant predictors (*F*(10, 189) = 1.17, *p* = 0.311, R^2^ = 0.058). Variance inflation factors were all below 2.0, indicating no multicollinearity. No sociodemographic covariate reached statistical significance in any model.

**Fig. 4.**
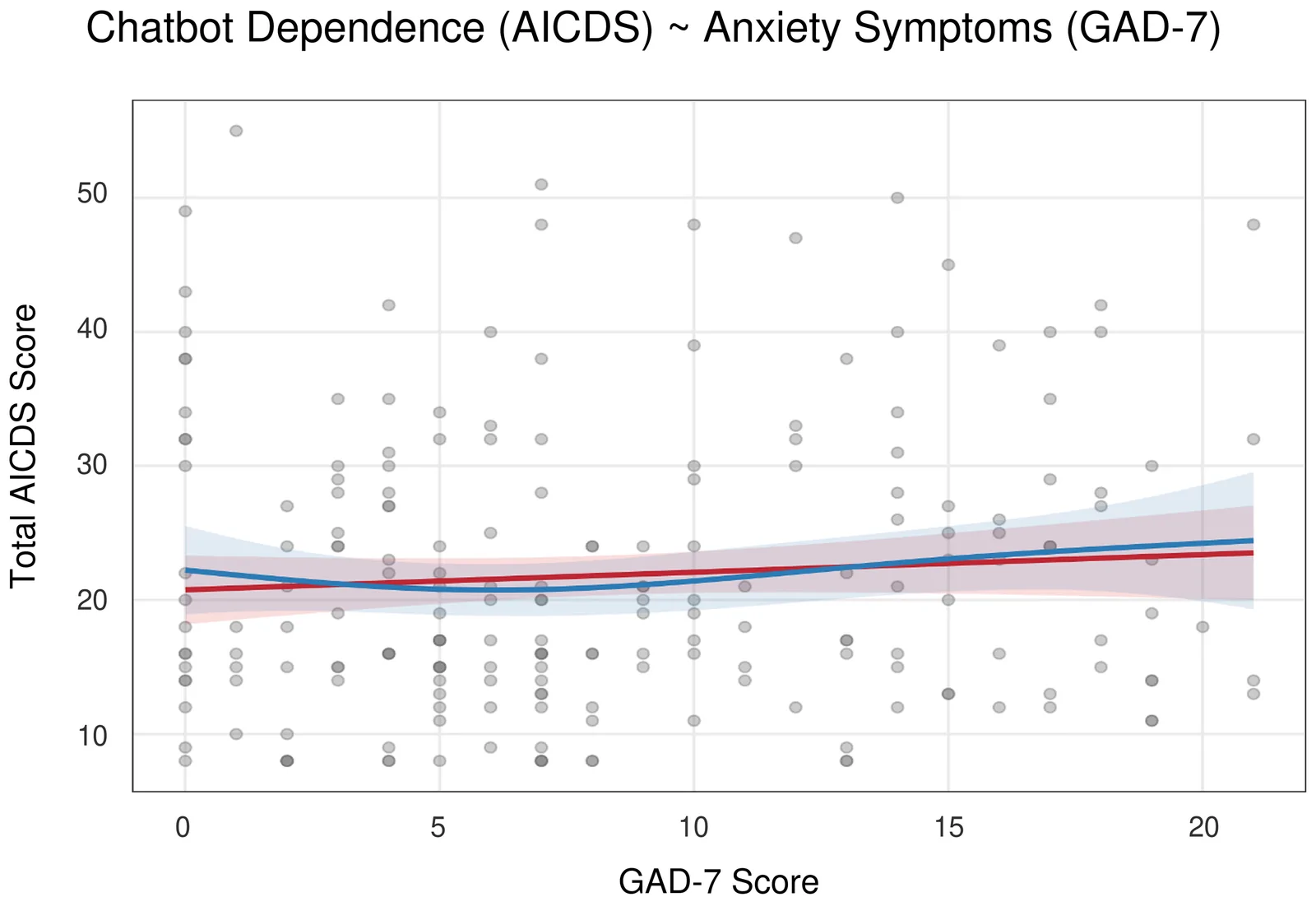
**Scatterplot of AICDS total score by GAD-7 score (N = 200)**. The red line represents the linear regression fit; the blue curve represents the generalized additive model (GAM) smooth (k = 5). The near-identical trajectories of both curves indicate no evidence of a nonlinear association.

**Fig. 5.**
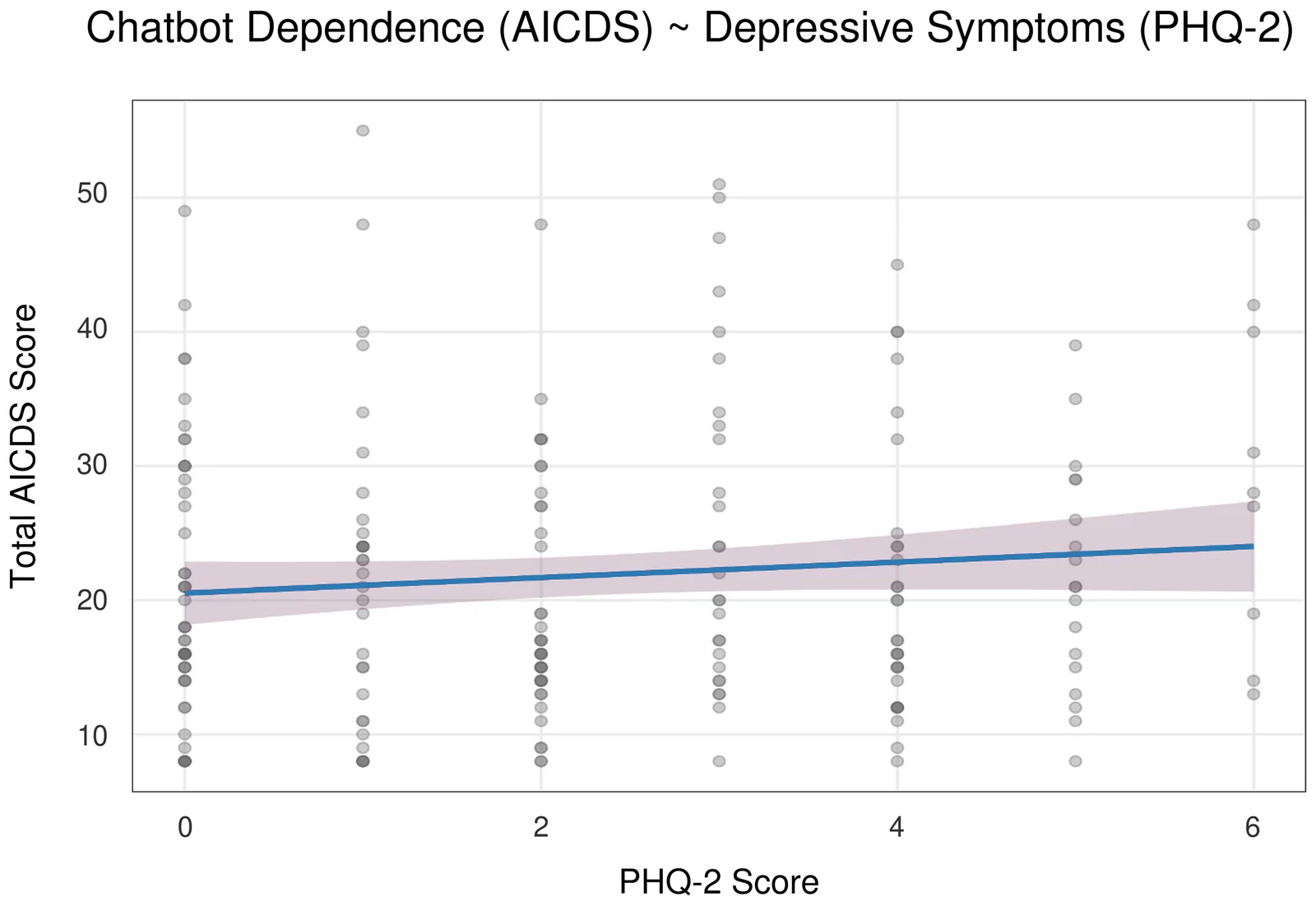
**Scatterplot of AICDS total score by PHQ-2 score (N = 200)**. The red line represents the linear regression fit; the blue curve represents the GAM smooth (k = 5). As with the GAD-7, both curves follow a near-identical trajectory, indicating a linear (and non-significant) association.

GAMs were fitted to test whether the associations between chatbot dependence and anxiety and depressive symptom scores were nonlinear. The smooth term for GAD-7 was not statistically significant, estimated degrees of freedom = 2.49, *F* = 1.95, *p* = 0.13, nor was the smooth term for PHQ-2, edf ≈ 1.00, *F* = 2.20, *p* = 0.14, indicating no evidence of nonlinear associations. Parametric quadratic models confirmed these findings; the addition of quadratic terms for GAD-7 and PHQ-2 did not significantly improve model fit, both *p* > 0.14. These patterns are also shown graphically in Figs. 4,5, where the associations between AICDS scores and both anxiety and depressive symptoms appear weak and approximately linear.

Exploratory moderation analyses examined whether field of study, sex, or primary purpose of chatbot use moderated the association between GAD-7 scores and chatbot dependence. No statistically significant interaction was identified for field of study, *F*(13, 186) = 1.24, *p* = 0.253, or sex, GAD-7 × sex interaction: B = 1.10, SE = 0.86, *p* = 0.201. Across the five purpose-of-use categories reported in Table 1 (resolving academic doubts, writing assignments, curiosity or entertainment, emotional support, and other), no statistically significant interaction with GAD-7 was identified. Given the small subgroup sizes within some categories, these analyses should be interpreted as exploratory. Overall, these analyses did not provide robust evidence that the association between anxiety symptoms and chatbot dependence differed meaningfully across demographic or use-related subgroups.

An exploratory mediation analysis tested whether anxiety symptoms (GAD-7) mediated the relationship between chatbot dependence and depressive symptoms (PHQ-2), using nonparametric bootstrap resampling (1000 iterations, BCa 95% CI). The average causal mediation effect (ACME) was small and non-significant (ACME = 0.009, 95% CI: −0.006 to 0.025, *p* = 0.238), as were the average direct effect (ADE = 0.009, *p* = 0.330) and total effect (0.018, *p* = 0.136). These findings suggest that anxiety does not mediate the relationship between chatbot dependence and depressive symptoms in this sample.

## 4. Discussion

### 4.1 Main Findings

To our knowledge, this study represents one of the first attempts in South America to examine AI chatbot dependence using a psychometric scale. The present study explored the relationship between dependence on artificial intelligence chatbots and symptoms of anxiety and depression among university students, while also examining the psychometric properties of the Spanish version of the AICDS.

The findings indicate that, in bivariate comparisons, higher chatbot dependence was associated with screening positive for anxiety symptoms. However, this association did not remain statistically significant after adjustment for sociodemographic covariates in multiple regression analyses. No statistically significant association was observed with depressive symptoms using two-tailed testing. In addition, the AICDS demonstrated good internal consistency and a coherent unidimensional factor structure, providing preliminary psychometric support for its use in assessing dependence on AI chatbot technologies among university students.

Within this sample, dependence levels did not significantly differ according to the sociodemographic or academic variables examined. This pattern may reflect the increasing integration of AI chatbots into academic routines across different student profiles, although broader generalizations require caution given the non-probabilistic sampling strategy and single-city setting.

From a psychometric perspective, the results provide preliminary evidence supporting the internal consistency and factorial validity of the Spanish version of the AICDS in this population. However, the CFA results should be interpreted cautiously [38,39]. Although CFI and TLI indicated strong fit and SRMR suggested adequate residual fit, the RMSEA value was at the upper boundary of the acceptable range. In addition, inspection of standardized residuals and modification indices suggested possible local dependence between some item pairs, particularly AICDS6 and AICDS7. Because there was no strong theoretical justification for adding correlated residuals, the original unidimensional model was retained. Taken together, these findings support the plausibility of a one-factor structure, but they should not be interpreted as full validation of the Spanish AICDS. Rather, they provide initial evidence of factorial validity in this specific sample and indicate the need for further psychometric evaluation in larger and more diverse populations.

### 4.2 Interpretation in Context of Literature

These findings align with emerging literature suggesting that artificial intelligence chatbots are increasingly incorporated into students’ academic routines as sources of rapid information, problem-solving assistance, and cognitive support [1,2]. Importantly, frequent use of AI chatbots should not be interpreted as inherently problematic. In educational contexts, repeated use may reflect legitimate academic engagement, AI literacy, self-regulated learning strategies, or pragmatic adaptation to new learning environments. The construct assessed by the AICDS is more specific, referring to perceived reliance, difficulty functioning without chatbot access, excessive trust, and repetitive consultation. Therefore, the present findings should be interpreted as preliminary evidence regarding a possible pattern of AI-related dependence, not as evidence that ordinary AI-assisted learning is maladaptive.

Previous research on technology-mediated behaviors has shown that excessive engagement with digital platforms, including internet use and social media, may be associated with psychological distress, sleep disruption, and emotional dysregulation among university students [14,20]. The present findings extend this line of research cautiously by suggesting that dependence-like patterns involving AI chatbots may represent an emerging domain of technology-related behavior that warrants further empirical and psychometric investigation.

The cultural adaptation of the AICDS is also relevant because Spanish-speaking university populations may differ in their educational practices, patterns of digital access, and meanings attributed to AI-assisted learning. In this manuscript, the term “Spanish version” refers to the language of the adapted instrument rather than to a sample from Spain. In Paraguay and other Latin American contexts, students may use AI chatbots not only as tools for academic productivity but also as accessible resources in settings where educational support, tutoring, or specialized academic guidance may be unevenly distributed. Therefore, the availability of a Spanish version of the AICDS may facilitate future research on AI-related behavioral patterns in regional contexts where evidence remains scarce. Nevertheless, the present findings should be interpreted as an initial step, and further studies are needed to examine semantic, cultural, and measurement equivalence across different Spanish-speaking countries and educational systems.

The additional analyses examining nonlinearity, moderation, and mediation should be interpreted as exploratory. These analyses were included to examine whether the weak bivariate association between anxiety screening status and AICDS scores could reflect more complex patterns; however, they did not provide evidence of nonlinear associations, subgroup-specific effects, or mediation by anxiety. In particular, the mediation analysis should be interpreted with caution because the study was cross-sectional and because the main associations between chatbot dependence, anxiety symptoms, and depressive symptoms were weak and not robust after adjustment. Therefore, these findings should not be understood as evidence of causal pathways. Instead, they suggest that, in this sample, AI chatbot dependence as measured by the AICDS was not strongly explained by general anxiety or depressive symptom severity.

### 4.3 AI Chatbot Use and Mental Health

The association observed between chatbot dependence and anxiety may reflect the role of these tools as mechanisms for reducing uncertainty and obtaining immediate reassurance. In academically demanding contexts, students frequently face cognitive overload, time pressure, and fear of making mistakes. Under such conditions, AI chatbots may function as rapid feedback systems that provide immediate answers and perceived academic support [41,42].

However, the constant availability of instant responses may reinforce reliance on external sources for decision-making and information verification. This dynamic may contribute to patterns of reassurance-seeking or avoidance of cognitive effort, particularly among individuals experiencing higher levels of anxiety. Similar mechanisms have been described in studies examining digital help-seeking behaviors and emotional regulation through online platforms [43,44].

Importantly, the cross-sectional nature of the present study does not allow conclusions regarding the direction of this relationship. It remains possible that students experiencing higher anxiety or depressive symptoms are more likely to rely on AI chatbots, rather than chatbot dependence causing distress.

### 4.4 Cognitive Offloading and Technological Dependence

Another possible explanation for the observed association involves the concept of cognitive offloading, defined as the tendency to rely on external tools or technologies to reduce cognitive effort or memory load. Cognitive offloading has been widely documented in relation to smartphones, digital reminders, and search engines [23,24,45].

AI chatbots may represent an advanced form of cognitive offloading because they provide not only information retrieval but also synthesized explanations, decision support, and problem-solving guidance. While these functions can enhance learning efficiency, excessive reliance may reduce active cognitive engagement or independent reasoning, potentially fostering psychological dependence on these technologies [46,47].

### 4.5 Implications for Higher Education and Mental Health

The findings have several implications for higher education institutions. As AI chatbots become increasingly integrated into academic practices, universities may need to consider how these technologies influence students’ learning processes and psychological well-being.

Educational policies should not necessarily discourage the use of AI tools, as they can serve valuable pedagogical functions. Instead, institutions may benefit from promoting balanced and reflective use of AI technologies, encouraging students to combine AI-assisted learning with critical thinking, independent analysis, and traditional study strategies. Furthermore, digital literacy programs may help students understand both the benefits and potential limitations of AI tools, thereby reducing the risk of excessive reliance [48,49]. Recent work on AI-supported university assessment has also highlighted the need to consider validity, reliability, and fairness when AI systems are incorporated into academic practices [50].

In practical terms, universities could incorporate AI literacy modules into first-year orientation or academic skills courses, emphasizing critical evaluation of chatbot outputs, appropriate citation and academic integrity practices, protection of personal data, and the importance of maintaining independent reasoning. Faculty members could also design assignments that require students to document how AI tools were used, compare AI-generated responses with scholarly sources, and reflect on the limitations of automated feedback. Such strategies may help preserve the pedagogical value of AI-assisted learning while reducing excessive or uncritical reliance on chatbot-generated content.

From a mental health perspective, the present findings do not support the use of chatbot dependence as a screening indicator for anxiety or depressive symptoms. The bivariate association with anxiety symptoms was small and did not remain statistically significant after covariate adjustment, while depressive symptoms were not significantly associated with AICDS scores under two-tailed testing. Therefore, any potential role of chatbot dependence as a marker of student distress should be considered only as a hypothesis for future research. Longitudinal studies with larger samples, predictive validity analyses, and clinically meaningful thresholds would be needed before any screening implication could be considered. Nevertheless, university counselling services and student well-being programs may benefit from remaining attentive to emerging patterns of AI-assisted learning and technology-related behavior as part of broader digital well-being initiatives [51,52]. At the same time, AI tools may also offer opportunities for mental health support. Conversational agents have been explored as tools for delivering psychoeducation, emotional support, and digital cognitive-behavioral interventions. For instance, randomized trials of AI-based chatbots such as Woebot have reported reductions in symptoms of depression and anxiety among young adults [53,54]. Future research should therefore examine how AI technologies may simultaneously represent potential sources of vulnerability and supportive resources for student well-being.

### 4.6 Strengths, Limitations and Future Directions

This study has several strengths. First, it contributes to a rapidly emerging area of research by examining the relationship between AI chatbot dependence and anxiety and depressive symptoms, a topic that has received limited empirical attention. Second, the study provides initial psychometric evidence for the Spanish version of the Artificial Intelligence Chatbot Dependence Scale, facilitating future research in Spanish-speaking populations. Third, the inclusion of validated mental health screening instruments (PHQ-2 and GAD-7) allowed the examination of associations between chatbot use and anxiety and depressive symptoms using established measures. Finally, the use of an appropriate estimation method (DWLS) for ordinal data strengthens the robustness of the psychometric analyses.

Several limitations should also be considered. The cross-sectional design prevents causal inference; therefore, it remains unclear whether greater chatbot dependence contributes to anxiety or depressive symptoms, or whether students experiencing higher anxiety or depressive symptoms are more likely to rely on AI chatbots. Furthermore, self-selection bias may have occurred because participation was voluntary and conducted through an online survey. Additionally, all measures relied on self-reported data collected at a single time point, which may increase the risk of common method bias, including social desirability, recall bias, and shared response tendencies across instruments. No procedural strategies such as temporal separation of measures, multi-informant data, or objective behavioral indicators were used, and no statistical test specifically designed to assess common method bias was applied. Therefore, the results should be interpreted with caution. The study was conducted in a single geographic location using a non-probabilistic online sampling strategy, and half of the sample came from medical and health sciences. Therefore, the findings should be interpreted as initial evidence from a specific university population in Santa Rosa del Aguaray, Paraguay, rather than as representative of Paraguayan or Spanish-speaking university students more broadly. In addition, anxiety and depression were assessed using screening instruments rather than clinical diagnostic interviews. Another limitation is that some potentially relevant confounding variables were not measured, including academic pressure, perceived social support, digital literacy, previous experience with AI tools, and general patterns of technology use. These factors may influence both chatbot dependence and anxiety or depressive symptoms, and their omission may have affected the precision and stability of the estimated associations. Finally, the relatively modest sample size and the absence of external validation or replication samples may limit the generalizability and stability of the factor structure.

Future research should explore these relationships using longitudinal designs to clarify potential causal pathways between AI chatbot dependence and mental health outcomes. Studies in diverse academic settings and cultural contexts would also be valuable to determine whether similar patterns are observed in other populations. Additionally, further psychometric evaluations of the AICDS are needed, including tests of measurement invariance across gender and academic disciplines, as well as test–retest reliability. Future studies should also examine convergent and discriminant validity using theoretically related constructs, such as problematic technology use, reassurance seeking, cognitive offloading, AI trust, and self-regulated learning, as well as unrelated constructs to better establish the boundaries of the AICDS. Cross-validation in independent samples would also be important.

## 5. Conclusions

In conclusion, this study provides preliminary evidence supporting the internal consistency and factorial validity of the Spanish version of the Artificial Intelligence Chatbot Dependence Scale in a sample of university students from Santa Rosa del Aguaray, Paraguay. Although students who screened positive for anxiety symptoms showed slightly higher chatbot dependence scores in bivariate analyses, this association was small and did not remain significant after adjustment. No robust association was observed with depressive symptoms. These findings suggest that the AICDS may be a useful instrument for future research on AI-related behavioral patterns, but additional validation studies with larger, more diverse, and longitudinal samples are needed before broader conclusions can be drawn about the mental health implications of AI chatbot dependence.

## Data Availability

The data supporting the findings of this study are available upon reasonable request to the corresponding author.

## References

[1] Morell-Mengual V, Fernández-García O, Berenguer C, Ortega-Barón J, Gil-Llario MD, Estruch-García V (2025). Characteristics, motivations and attitudes of students using ChatGPT and other language model-based chatbots in higher education. Education and Information Technologies.

[2] Salsabilla D, Kesuma ME, Gunawan I, Ashari A (2025). The Use of Artificial Intelligence Chatbots on Student Learning, Information, and Communication in Higher Education. KnE Social Sciences.

[3] Kasneci E, Seßler K, Küchemann S, Bannert M, Dementieva D, Fischer F (2023). ChatGPT for good? On opportunities and challenges of large language models for education. Learning and Individual Differences.

[4] Torales J, O'Higgins M (2024). ChatGPT and social psychiatry: A commentary on the article 'Old dog, new tricks? Exploring the potential functionalities of ChatGPT in supporting educational methods in social psychiatry'. The International Journal of Social Psychiatry.

[5] Torales J, O'Higgins M, Di Martino B, Kunihiro D, Carbajal L, Pereira F (2026). ChatGPT-4 in Psychiatric Discharge Summaries: Toward a More Comprehensible and Emotionally Sensitive Clinical Communication. The International Journal of Social Psychiatry.

[6] Zawacki-Richter O, Marín VI, Bond M, Gouverneur F (2019). Systematic review of research on artificial intelligence applications in higher education–where are the educators?. International Journal of Educational Technology in Higher Education.

[7] Adamopoulou E, Moussiades L (2020). An overview of chatbot technology.

[8] Torales J, Torres-Romero AD, Di Giuseppe MF, Rolón-Méndez ER, Martínez-López PL, Heinichen-Mansfeld KV (2022). Technostress, anxiety, and depression among university students: A report from Paraguay. The International Journal of Social Psychiatry.

[9] Lei X, Liu C, Jiang H (2021). Mental health of college students and associated factors in Hubei of China. PloS One.

[10] Wang C, Yan S, Jiang H, Guo Y, Gan Y, Lv C (2022). Socio-demographic characteristics, lifestyles, social support quality and mental health in college students: a cross-sectional study. BMC Public Health.

[11] Auerbach RP, Mortier P, Bruffaerts R, Alonso J, Benjet C, Cuijpers P (2018). WHO World Mental Health Surveys International College Student Project: Prevalence and distribution of mental disorders. Journal of Abnormal Psychology.

[12] Ibrahim AK, Kelly SJ, Adams CE, Glazebrook C (2013). A systematic review of studies of depression prevalence in university students. Journal of Psychiatric Research.

[13] Ochnik D, Rogowska AM, Kuśnierz C, Jakubiak M, Schütz A, Held MJ (2021). Mental health prevalence and predictors among university students in nine countries during the COVID-19 pandemic: a cross-national study. Scientific Reports.

[14] Radovanovic J, Selakovic V, Mihaljevic O, Djordjevic J, Čolović S, Djordjevic JR (2023). Mental health status and coping strategies during COVID-19 pandemic among university students in Central Serbia. Frontiers in Psychiatry.

[15] Aristovnik A, Keržič D, Ravšelj D, Tomaževič N, Umek L (2020). Impacts of the Covid-19 Pandemic on Life of Higher Education Students: A Global Perspective. Sustainability.

[16] Cao W, Fang Z, Hou G, Han M, Xu X, Dong J (2020). The psychological impact of the COVID-19 epidemic on college students in China. Psychiatry Research.

[17] Son C, Hegde S, Smith A, Wang X, Sasangohar F (2020). Effects of COVID-19 on College Students' Mental Health in the United States: Interview Survey Study. Journal of Medical Internet Research.

[18] Figueredo P, Barrios I, O'Higgins M, Amarilla D, Almirón-Santacruz J, Melgarejo O (2022). Anxiety, Addiction to Social Networks, Internet and Smartphones in Paraguayan Adolescents: A Brief Report. Scandinavian Journal of Child and Adolescent Psychiatry and Psychology.

[19] Kuss DJ, Griffiths MD (2017). Social Networking Sites and Addiction: Ten Lessons Learned. International Journal of Environmental Research and Public Health.

[20] Wang Y, Zhao Y, Liu L, Chen Y, Ai D, Yao Y (2020). The Current Situation of Internet Addiction and Its Impact on Sleep Quality and Self-Injury Behavior in Chinese Medical Students. Psychiatry Investigation.

[21] Zhu W, Zhang Y, Lan Y, Song X (2025). Smartphone dependence and its influence on physical and mental health. Frontiers in Psychiatry.

[22] Giuntella O, Konig J, Stella L (2025). Artificial intelligence and the wellbeing of workers. Scientific Reports.

[23] Risko EF, Gilbert SJ (2016). Cognitive Offloading. Trends in Cognitive Sciences.

[24] Sparrow B, Liu J, Wegner DM (2011). Google effects on memory: cognitive consequences of having information at our fingertips. Science (New York, N.Y.).

[25] Zhang X, Yin M, Zhang M, Li Z, Li H (2025). The Development and Validation of an Artificial Intelligence Chatbot Dependence Scale. Cyberpsychology, Behavior and Social Networking.

[26] Beaton DE, Bombardier C, Guillemin F, Ferraz MB (2000). Guidelines for the process of cross-cultural adaptation of self-report measures. Spine.

[27] Boateng GO, Neilands TB, Frongillo EA, Melgar-Quiñonez HR, Young SL (2018). Best Practices for Developing and Validating Scales for Health, Social, and Behavioral Research: A Primer. Frontiers in Public Health.

[28] Setia MS (2016). Methodology Series Module 3: Cross-sectional Studies. Indian Journal of Dermatology.

[29] Gosling SD, Vazire S, Srivastava S, John OP (2004). Should we trust web-based studies? A comparative analysis of six preconceptions about internet questionnaires. The American Psychologist.

[30] Kroenke K, Spitzer RL, Williams JBW (2003). The Patient Health Questionnaire-2: validity of a two-item depression screener. Medical Care.

[31] Torales J, Barrios I, Ayala N, O’Higgins M, Palacios JM, Ríos-González C (2021). Ansiedad y depresión en relación a noticias sobre COVID-19: un estudio en población general paraguaya. Revista de salud pública del Paraguay.

[32] Spitzer RL, Kroenke K, Williams JBW, Löwe B (2006). A brief measure for assessing generalized anxiety disorder: the GAD-7. Archives of Internal Medicine.

[33] García-Campayo J, Zamorano E, Ruiz MA, Pardo A, Pérez-Páramo M, López-Gómez V (2010). Cultural adaptation into Spanish of the generalized anxiety disorder-7 (GAD-7) scale as a screening tool. Health and Quality of Life Outcomes.

[34] Hair JF, Black WC, Babin BJ, Anderson RE (2019). Multivariate data analysis.

[35] Kline RB (2016). Principles and practice of structural equation modeling.

[36] Kyriazos TA (2018). Applied psychometrics: sample size and sample power considerations in factor analysis (EFA, CFA) and SEM in general. Psychology.

[37] White M (2022). Sample size in quantitative instrument validation studies: A systematic review of articles published in Scopus, 2021. Heliyon.

[38] Li CH (2016). The performance of ML, DWLS, and ULS estimation with robust corrections in structural equation models with ordinal variables. Psychological Methods.

[39] Xia Y, Yang Y (2019). RMSEA, CFI, and TLI in structural equation modeling with ordered categorical data: The story they tell depends on the estimation methods. Behavior Research Methods.

[40] Hu LT, Bentler PM (1999). Cutoff criteria for fit indexes in covariance structure analysis: Conventional criteria versus new alternatives. Structural Equation Modeling: A Multidisciplinary Journal.

[41] Karayianni I, Klidas A, Karakitsou C (2026). Between a bot and a hard place: student engagement, technology anxiety and ChatGPT trust in higher education. TechTrends.

[42] Kohler K (2024). You only need to change your direction: A look at the potential impact of ChatGPT on education. Technology in Language Teaching & Learning.

[43] Shi K, Tian J (2025). The impact of mental health literacy on professional psychological help-seeking attitudes among Chinese college students: the chain mediating role of anxiety and depression. Frontiers in Psychology.

[44] Ye X, Gao H (2025). Distress disclosure on social media and depressive symptoms among college students: the roles of social comparison and gender. Frontiers in Psychology.

[45] Storm BC, Stone SM (2015). Saving-enhanced memory: the benefits of saving on the learning and remembering of new information. Psychological Science.

[46] Jose B, Cherian J, Verghis AM, Varghise SM, S M, Joseph S (2025). The cognitive paradox of AI in education: between enhancement and erosion. Frontiers in Psychology.

[47] Zhang Y (2025). GPT is all you need. Frontiers in Psychology.

[48] Atchley P, Pannell H, Wofford K, Hopkins M, Atchley RA (2024). Human and AI collaboration in the higher education environment: opportunities and concerns. Cognitive Research: Principles and Implications.

[49] Grinschgl S, Neubauer AC (2022). Supporting Cognition With Modern Technology: Distributed Cognition Today and in an AI-Enhanced Future. Frontiers in Artificial Intelligence.

[50] Zacharis G, Papadakis S (2025). Can AI Grade Like a Human? Validity, Reliability, and Fairness in University Coursework Assessment. Educational Process: International Journal.

[51] Humayun A, Madawana AM, Hassan A, Mahmud A, Kamaruddin N, Noor SH (2025). Artificial intelligence as a predictive tool for mental health status: Insights from a systematic review and meta-analysis. PloS One.

[52] Kim TW, Usman U, Garvey A, Duhachek A (2026). From algorithm aversion to AI dependence: Deskilling, upskilling, and emerging addictions in the GenAI age. Consumer Psychology Review.

[53] Fitzpatrick KK, Darcy A, Vierhile M (2017). Delivering Cognitive Behavior Therapy to Young Adults With Symptoms of Depression and Anxiety Using a Fully Automated Conversational Agent (Woebot): A Randomized Controlled Trial. JMIR Mental Health.

[54] Inkster B, Sarda S, Subramanian V (2018). An Empathy-Driven, Conversational Artificial Intelligence Agent (Wysa) for Digital Mental Well-Being: Real-World Data Evaluation Mixed-Methods Study. JMIR MHealth and UHealth.

